# Evaluation of the xerostomia, taste and smell impairments after Covid-19

**DOI:** 10.4317/medoral.24510

**Published:** 2021-03-27

**Authors:** Mehmet Melih Omezli, Damla Torul

**Affiliations:** 1 Department of Oral and Maxillofacial Surgery, Faculty of Dentistry, Ordu University, Ordu, Turkey

## Abstract

**Background:**

The purpose of this study was to explore the effects of coronavirus disease 19 (Covid-19) on the oral cavity by evaluating the oral findings in the patients who recovered after treatment.

**Material and Methods:**

This study involved confirmed Covid-19 patients whose treatment completed at least two weeks ago. A questionnaire consist of eight parts was applied to explore the oral findings after Covid-19. Also stimulated salivary flow rate was evaluated with a salivary flow test.

**Results:**

177 patients reached and 107 of them participate in the study. Regarding gender significant differences were found in terms of the presence of taste impairment after treatment (*p*=0.007), the degree of taste (*p*=0.021) and smell (*p*=0.010) impairment. 18 % (5/27) of the patients evaluated were showed hyposalivation. No significant differences were observed regarding salivary flow between males (mean±SD: 1.14±0.65) and females (mean±SD: 1.12±0.43), (*p*=0.928); among the patients having treatment at home (mean±SD: 1.03±0.48) or hospital (mean±SD: 1.33±0.65), (*p*=0.187). In some of the patients’ taste [15], smell [23] impairment, and xerostomia [43] still observed at least two weeks after the treatment is completed.

**Conclusions:**

The most frequent finding in patients after the treatment was xerostomia. Taste and smell impairments were more frequently observed in females.

** Key words:**Saliva, oral findings, hyposalivation, Covid-19.

## Introduction

In late 2019, a new type of coronavirus named 2019 novel coronavirus (2019-nCoV) has emerged in China and caused a global burden in a short period of time (1,2). Studies related to the pathogenesis of this virus reported that the virus enters the host cells with spike (S) protein by binding to the angiotensin converting enzyme II (ACE 2) receptors with high affinity (3,4). ACE-2 receptor reported being expressed in nearly 70 human tissues (1,5). ACE-2 express in the oral cavity mainly in epithelial cells of tongue and oral mucosa (6). The expression of ACE-2 in the salivary glands has also been reported (7,8). Therefore, it is considered that the oral mucosa, and salivary glands may be a potential target for 2019-nCoV.

The clinical manifestations of coronavirus disease 19 (Covid-19) majorly non-specific and include shortness of breath, fever, muscle pain, cough, sputum, arthralgia, diarrhea, rhinorrhea, headache and sore throat reported by the clinical studies from Asia (9-11). However, with the spread of the infection on the European continent, atypical new symptoms of the disease such as smell and taste disturbance have emerged. The frequency of taste and smell disturbance was reported in different studies between 19.4 % and 88 % in the first coronavirus cases in Europe (9,12-14). Beside manifest clinically with a variety of symptoms, it is also argued based on the similarities with severe acute respiratory syndrome coronavirus (SARS-CoV) that 2019-nCoV may lead in some instance permanent damage associated with an excessive immune reaction in the host cells (2,15,16).

The consequences of the Covid-19 in patients who were survived after treatment is not clearly understood with limited information regarding different systems of the body present in the literature (17-19). To our knowledge there is no study in the literature explore the oral findings of the patients who recovered after treatment. The aim of this study is therefore to investigate the oral findings in the patients who survived after Covid-19.

## Material and Methods

This study was carried out between June 14 and October 14, 2020 with the survivors of confirmed Covid-19 who had received treatment in the hospital or had medications and followed in-home quarantine.

- Inclusion Criteria

1. Patients who were volunteer to participate,

2. Patients who were confirmed Covid-19 with PCR and discharged at least 2 weeks ago

3. Patients who did not have any comorbid disease that would affect the saliva flow/amount (Sjögren's syndrome, rheumatoid arthritis, systemic lupus erythematosus, sarcoidosis, tuberculosis, graft versus host disease, cystic fibrosis, bell palsy, uncontrolled diabetes, amyloidosis, Alzheimer’s disease, HIV infection, hepatitis C, hypo and hyperthyroidism, anorexia, bulimia)*

4. Patients who did not use any medication that would affect the saliva flow/amount (anorexiants, antiacne, anticholinergic/antispasmodic, anticonvulsives, antidepressants, antihistamines, antihypertensives, non-steroidal anti-inflammatory analgesics, antiemetics, antiparkinson, antipsychotics, bronchodilators, chemotherapy agents, decongestants, diuretics, muscle relaxants, narcotic analgesics, and sedative agents)*

5. Patients who had no salivary gland disease or operation for any reason before Covid-19*

6. Patients who did not have radiotherapy*

* Additional inclusion criteria for the participants who also included in salivary flow test

- Exclusion Criteria

1. Patients who did not want to participate,

2. Patients who have a taste and/or smell alteration before Covid-19,

3. Patients who were unable to complete online questionnaire.

4. Patients who have systemic comorbidities or use medication that affect the salivary flow/amount,**

5. Patients who were unable to collect saliva**.

** Exclusion criteria from saliva flow test

**Figure 1:**
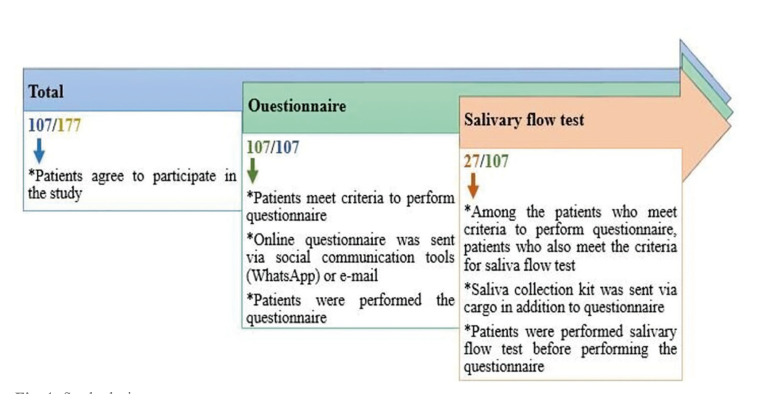
Study design.

Patients contacted by email and social communication tools (WhatsApp application etc) and given information about the study. The individuals who respond and declare to be a volunteer to participate in the study were sent a questionnaire link prepared via Google forms (Google LLC, Mountain View, California) to evaluate whether there are changes in oral health after Covid-19 via social communication tools (WhatsApp) or e-mail. They were asked to complete the questionnaire. The individuals who meet inclusion criteria for salivary flow test were further sent a saliva collection kit (instructions, sterile millimetric striped tube, glove, and sugar-free gum) via cargo. They were asked to collect their saliva according to the instructions sent with the kit. The study design was shown in Fig. 1.

The patients supervised during saliva collection by the same researcher through appropriate communication tools (Mobil phone, video call) to ensure the standardization of the collection procedure. All patients were asked to perform the saliva collection between 11:00 and 12:30 am after breakfast. Patients were advised not to eat, drink, chew gum, or brush their teeth for at least 1 hour before collecting saliva. Prior to saliva collection patients wanted to first rinse their mouth with water for 30 seconds. Then, while sitting, they were asked to chew the sugar-free gum for 1-2 minutes and swallow the saliva formed during this time. Subsequently, they were asked to continue chewing the sugar-free chewing gum 50-60 times/minute, for 5 minutes and constantly split the formed saliva in a sterile tube during this period. The amount of saliva collected in tubes was determined by reading the level of collected saliva through the millimetric striped tube. The flow rate was calculated as ml/min. According to the saliva flow index the stimulated and unstimulated saliva flow classified as normal, low, or very low (hyposalivation). In adults, the normal total stimulated saliva flow ranges from 1 to 3 ml/min. Hyposalivation occurs when the salivary flow is less than 0.7 ml/min for stimulated saliva. Thus, the flow rate was below 0.7 ml/min evaluated as reduced stimulated saliva production (20-22).

- Questionnaire

All patients were queried on oral findings, using a questionnaire. The questionnaire consists of eight parts. The first part assessed the patients' demographic information (gender, age, education, systemic diseases, medications used, and smoking-alcohol consumption). The second part evaluates treatment type, and treatment duration in hospital. The third and fourth parts evaluate taste-smell impairment. The last three parts were evaluated xerostomia/ stimulated salivary flow rate, burning mouth, and pain on chewing, respectively. The taste and smell impairment, and need to sip liquid during eating scored as present and absent, pain on chewing scored as never, rarely, mostly. A 0 to 3-point scale (from 0 = absent to 3 = severe) used to evaluate degree of taste and smell impairment. A 0 to 5-point scale (from 0 = absent to 5 = severe) was used to evaluate xerostomia and burning mouth. The survey data were obtained from the google forms.

- Statistical analysis

Statistical analyses were performed using IBM SPSS Statistics for Windows software (version 23.0, IBM Corp, Chicago, USA). Kolmogorov Simirnov and Shapiro-Wilk tests were used to explore the normality of the continuous variables. Continuous data were expressed as Mean± (SD) and Median (Min-Max), categorical variables present as frequencies. Mann-Whitney U test and independent samples t tests were used to compare continuous variables as appropriate. For categorical variables Chi Square test was used. All tests were two-tailed, and *p*< 0.05 were accepted as significant.

## Results

 A total of 177 patients were reached and 107 patients (51 females, 56 males) between 13 and 70 years old with a mean age of 34.532±11.08 were agreed to participate and completed the questionnaire (60.45%). 27 of the 107 patients were also completed the salivary flow test. All participants completed the questionnaire and salivary flow test 2 to 4 weeks after discharged. The descriptive of the patients were shown in Table 1.

In terms of age no significant difference was observed between patients having treatment at home or hospital (*p*=0.174). No significant differences were found in terms of change in taste perception (*p*=0.404, *p*=0.436) and smell (*p*=0.345, *p*=0.234) either during and after treatment respectively, and pain on chewing (*p*=0.269) after treatment among the patients having treatment at home or hospital. No significant differences also found between patients having treatment at home or hospital in terms of the degree of taste (*p*=0.970) and smell (*p*=0.877) impairment, xerostomia (*p*=0.128), burning mouth (*p*=0.219), and need to sip liquid during eating solid foods (*p*=0.107).

No significant difference was observed between male and female patients (*p*=0.585) in terms of age. No significant difference was found in terms of change in taste perception during treatment (*p*=0.086) while a significant difference was observed after treatment (*p*=0.007). Regarding smell, no significant differences were found (*p*=0.131, *p*=0.057) either during and after treatment respectively, between the male and female patients. Significant differences were also found between male and females in terms of the degree of taste (*p*=0.021) and smell (*p*=0.010) impairment. No significant differences were observed in terms of xerostomia (*p*=0.233), burning mouth (*p*=0.650), need to sip liquid during eating solid foods (*p*=0.118), and pain on chewing (*p*=0.517) after treatment (Table 2, Table 3).

No significant differences were observed regarding the amount of saliva between males (mean±SD: 1.14±0.65) and females (mean±SD: 1.12±0.43), (*p*=0.928). No significant differences were observed regarding the amount of saliva among the patients having treatment at home (mean±SD: 1.03±0.48) or hospital (mean±SD: 1.33±0.65), (*p*=0.187). Only 5 of the 27 patients show a salivary flow rate below 0.7ml/min. Persistent taste and smell impairment observed in 15 and 23 patients, respectively. Need to sip liquid during eating solid foods, pain on chewing, burning mouth and xerostomia reported in 50, 18, 15, 43 patients respectively at least two weeks after the treatment completed (Table 4).

**Table 1:**
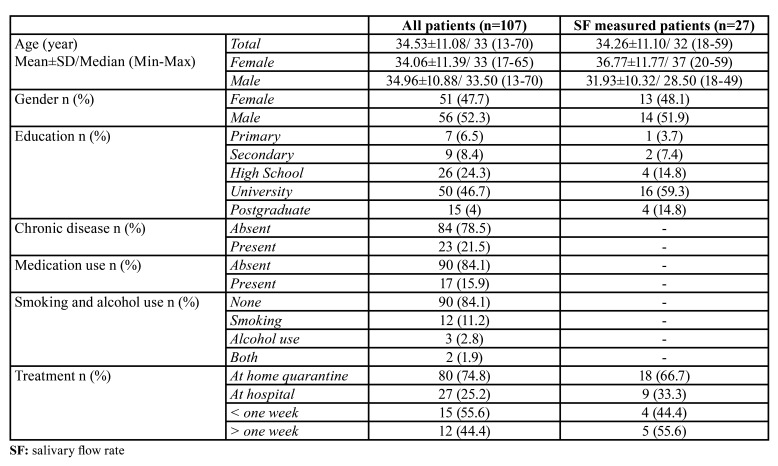
Descriptive of the patients.

**Table 2:**
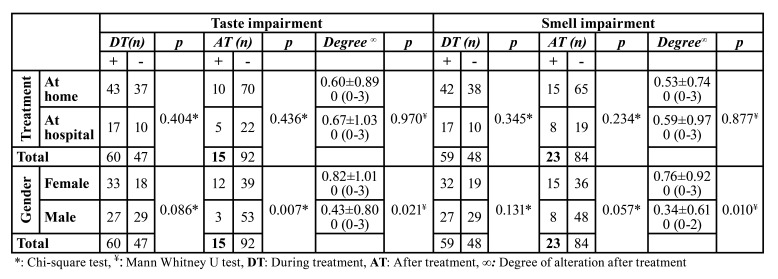
Taste and smell functions’ changes among patients.

**Table 3:**
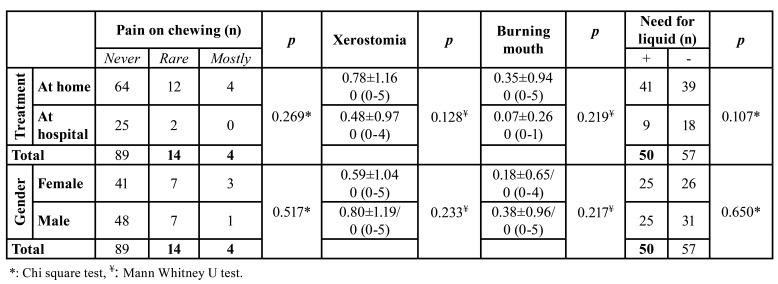
Distribution of oral findings at least two weeks after treatment complete.

**Table 4:**
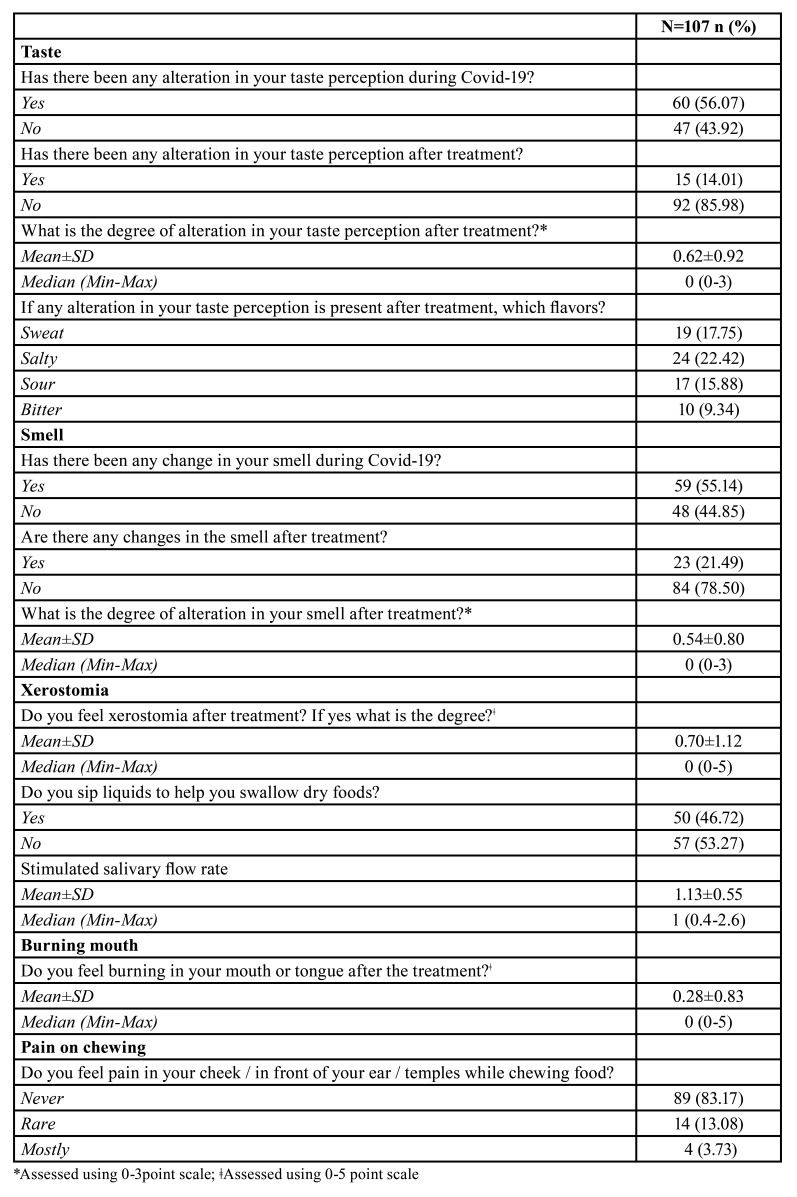
Patients reported oral findings.

## Discussion

 Since the emergence of the Covid-19 pandemic more attention has been paid to the pathogenesis, symptoms, diagnosis, prevention strategies and treatment of disease. However, with the increase in the number the patients who survived long term consequences of the infection on different systems become an area of concern. In this study, we aimed to determine the consequences of Covid-19 which occur in the oral cavity of the Covid-19 patients after treatment. We have reached 107 patients either treated at home or hospital and whose treatment completed at least 14 days ago. In terms of the results of the questionnaire most of the patients each treated at home or hospital reported taste impairment during the disease period but after the treatment majority of the patients reported that the taste and smell get back to normal function. Persistent alteration observed in 15 patients in terms of taste and 23 patients in smell function at least two weeks after the treatment is completed. The impairment in taste and smell function was significantly higher in females when compared with the males.

In the study of Lechien *et al*. (9) on 417 mild-moderate stage Covid-19 patients, it is reported that in nearly 25 % of the patients smell and taste functions recovered 2 weeks after symptoms resolved and they estimated that 56 % of the patients have persistent smell dysfunction following the resolution of the Covid-19 symptoms. Also, females in this study were significantly showed more impairment regarding smell and taste functions than males. In the study of Hopkins *et al*. (23) who performed initial and follow-up questionnaires to explore smell and taste disturbance suggested that 17.3 % of the patients report persistent complete loss of smell function, 1 to 4 weeks period. In another study Giacomelli *et al*. (14) interviewed 59 hospitalized Covid-19 patients and found that 34 % of patients reported smell or taste disturbance with females more frequently than males. Similarly, Lee *et al*. (24) observed that loss of smell or taste function was significantly more common among females. Also, they observed that smell or taste impairment persist for approximately 40 days in some patients. In a recent systematic review prevalence of taste impairment in patients with Covid-19 reported as 45 % with females found to be more affected. Although not clearly demonstrated this considered to be originating from that the hormonal modulation and exacerbated immune response in females contribute to higher dysfunction (25). In this study similar to the other studies published previously we found that females reported impairment in taste and smell more frequently and with higher severity than males.

Because of the similarities with SARS-CoV long term consequences also expected for Covid-19. Persisted smell disturbance that continued for two years was reported by Hwang (26) after SARS infection. In this study, most of the patients taste and smell function impairment recovered after treatment however in some of the patients taste and smell function still did not recover at least two weeks after treatment. The present study evaluates up to a one-month period after treatment, thus, patients with persistent impairment regarding taste and smell function in this study are required follow-up to determine if the Covid-19 leads to persistent or permanent loss of taste and smell function in these patients.

Although not clearly demonstrated, the 2019-nCoV infection is considered to affect the oral tissues in several ways. The inflammatory response occurs during the Covid-19 may lead damage in the oral tissues like epithelium, salivary glands, the tongue which are rich in ACE-2 receptor. If this permanent damage occurs in the salivary gland tissues this may result in hyposalivation (2). Saliva has a crucial role in oral health maintenance. Decreased salivary flow causes oral discomfort, increased caries risk, susceptibility to co-infections, altered taste sensation, difficulties with speech, mastication and swallowing (27,28). Because a specific pharmacotherapy has not been determined for Covid-19 yet, a multi-drug treatment used in 2019-nCoV patients. It is also believed that the side effects of these drugs like stomatitis, oral ulcers, dry mouth, reduced saliva, deterioration of the oral microbiota, opportunistic fungal infections, recurrent oral herpes infection negatively affect oral health. In addition, topical antiseptic applications, stress and poor oral hygiene, could also induce oral lesions. However, it is still not clear whether the oral manifestations resulting from the direct effect of 2019-nCoV infection or originated from immune system impairment, coinfections, and side effects of medications (25,29,30).

Oral findings like xerostomia, oral ulcers, pain in function reported in the literature appears to be in different oral regions like palate, gingiva, lip and tongue during the infection (31). In the study of Chen *et al*. (7) who conducted a survey about the oral symptoms of 108 Covid-19 patients it is reported that xerostomia and amblygeustia observed the majority of the patients. Brandao *et al*. (16) argued that the interaction between 2019-nCoV and ACE2 might cause disturbance on the keratinocytes function and the epithelial cells of the salivary glands ducts, and lead to oral lesions. Although oral manifestations of the Covid-19 reported in different studies in the literature to the best of our knowledge there is no study that explored the oral findings of the Covid-19 patients after recovery. In this study to minimize the effect of the medications used during the treatment we evaluate the patients who completed their treatment at least 14 days ago. Also, we only evaluate the amount of stimulated saliva in the patients who did have any comorbidity or use medication which affects the normal salivary flow. In the patients who the salivary flow evaluated only 5 of 27 patients show salivary flow below the level of 0.7 ml/min. This result may be caused by that the only mild-moderate patients could be evaluated in this study. Thus it still needs to be determined the salivary flow in the patients who have severe disease and treated in the intensive care unit. Among the 107 patients evaluated, 50 patients reported needing sip a liquid during eating solid foods, 43 patients reported xerostomia, 18 patients reported feeling pain on chewing, and 15 patients reported burning mouth in different severities at least 14 days after treatment.

The study has some limitations. First, the sample size is small and includes mild-moderate patients with few co-morbidities and good prognosis, thus cannot represent general Covid-19 patients. However, most of the Covid-19 survivors reached did not want to participate in the study. We think that this may be originated from the psycho-social effects of the disease. Second the salivary flow before the infection and other factors like stress, that may affect the health of the oral cavity could not have been evaluated. The questionnaire used was self prepared and is a subjective way to evaluate the oral findings in these patients. Also, although supervised by the researcher self-performed sialometry is another limitation.

Because of the multifactor effects in question on oral health during the infection period the exact mechanism that triggers the disturbance of oral health remains to be elucidated with more evidence during the infection period and also in the long term. In this study in some patients who survived taste and smell impairment, xerostomia, burning mouth and pain on chewing still observed at least 14 days after they completed their treatment. Thus, after treatment of Covid-19 more attention should be paid to the status of oral health including salivary glands. It may be useful to perform oral care monitoring for Covid-19 survivors to observe the long term effects of Covid-19 disease and also maintain oral health.
